# Prenatal Maternal Anxiety as a Risk Factor for Preterm Birth and the Effects of Heterogeneity on This Relationship: A Systematic Review and Meta-Analysis

**DOI:** 10.1155/2016/8312158

**Published:** 2016-05-19

**Authors:** M. Sarah Rose, Gianella Pana, Shahirose Premji

**Affiliations:** ^1^Research Facilitation, Alberta Health Services, Calgary, AB, Canada T2N 2T9; ^2^Faculty of Medicine, University of Calgary, AB, Canada T2N 1N4; ^3^Faculty of Nursing and Cumming School of Medicine, Department of Community Health Sciences, University of Calgary, AB, Canada T2N 1N4

## Abstract

*Background.* Systematic reviews (SR) and meta-analyses (MA) that previously explored the relationship between prenatal maternal anxiety (PMA) and preterm birth (PTB) have not been comprehensive in study inclusion, failing to account for effects of heterogeneity and disagree in their conclusions.* Objectives.* This SRMA provides a summary of the published evidence of the relationship between PMA and PTB while examining methodological and statistical sources of heterogeneity.* Methods.* Published studies from MEDLINE, CINAHL, PsycINFO, and EMBASE, until June 2015, were extracted and reviewed.* Results.* Of the 37 eligible studies, 31 were used in this MA; six more were subsequently excluded due to statistical issues, substantially reducing the heterogeneity. The odds ratio for PMA was 1.70 (95% CI 1.33, 2.18) for PTB and 1.67 (95% CI 1.35, 2.07) for spontaneous PTB comparing higher levels of anxiety to lower levels.* Conclusions.* Consistent findings indicate a significant association between PMA and PTB. Due to the statistical problem of including collinear variables in a single regression model, it is hard to distinguish the effect of the various types of psychosocial distress on PTB. However, a prenatal program aimed at addressing mental health issues could be designed and evaluated using a randomised controlled trial to assess the causal nature of different aspects of mental health on PTB.

## 1. Introduction

Preterm birth (PTB), commonly defined as delivery that occurs at a gestational age less than 37 weeks, poses a public health concern since critically underdeveloped infants are at a higher risk for neonatal mortality and survivor morbidity [1–3]. Preterm infants require longer hospital stays and are hospitalized more often as they are at risk for major health complications in infancy, development, and paediatric problems through childhood and chronic diseases in adulthood [3, 4]. Substantial attention has been paid to the role of prenatal maternal mental health problems in the aetiology of PTB. Theoretical models have been developed to explain the biological effect of prenatal maternal mental health problems, such as the physiological stress response of the hypothalamic-pituitary axis (HPA) regulated by corticotrophin-releasing hormone (CRH) [5, 6]. The pathways by which maternal mental health problems initiate a physiologic sequence of events that promote early labour, however, remain unknown [2, 5, 6].

Maternal mental health is a state of well-being in which a mother can cope and work productively against life stressors [4]. Maternal mental health problems include depression, anxiety, and stress. The relationship between prenatal maternal anxiety and PTB has been examined previously (SR). Two broad narrative reviews on the hypothesized and known mechanistic effects of stress on preterm labour concluded that the strongest predictor of PTB was pregnancy-specific anxiety [3, 7]. Although efficient and informative, such reviews are subject to selection bias [8]. There have been two SR with meta-analysis (SRMA) [9, 10] that focused on the relationship between prenatal maternal anxiety during pregnancy and PTB with conflicting results. Ding et al. [9] found that prenatal maternal anxiety was significantly associated with an increased risk for PTB and remained significant regardless of the timing of anxiety assessment. In contrast, Littleton et al. [10] reported nonsignificant summary correlation coefficients between anxiety during pregnancy and gestational age at birth and between pregnancy-specific anxiety and gestational age at birth. Explicit criteria for selecting and critically appraising the primary research studies were not always evident in these reviews. Inconsistencies in the findings of the SRMA and primary studies examining the relationship between anxiety and PTB may have also arisen from potential source of heterogeneity, such as differences in the primary predictor variable measured (type of anxiety), how the predictor variable is measured, and how the outcome is determined, to name only a few. The present study was designed to be a more inclusive and comprehensive SR and MA than previous studies and the goal was to determine the effect of potential sources of heterogeneity on the relationship between PTB and anxiety, which may help to explain conflicting evidence.

The overall aim of this SR and MA is to provide a summary of the peer-reviewed published evidence regarding the relationship between maternal anxiety during pregnancy and PTB, after accounting for several potential sources of heterogeneity. The specific objectives are (1) to determine sources of heterogeneity in the methodology and analysis of the studies, (2) to assess which of the sources have an impact on the estimation of the relationship of interest, and (3) to estimate the combined effect of studies within homogenous subgroups of studies.

## 2. Methods

### 2.1. Definitions

Prenatal maternal anxiety can be subdivided into three different types: trait anxiety (TA), state anxiety (SA), and pregnancy-specific anxiety (PSA). TA refers to the mother's relatively stable propensity for anxiety whereas SA refers to the temporary anxious feeling the mother develops due to a stressful event, which may or may not be related to her pregnancy [11]. PSA is then considered the mental state of a pregnant woman whose concerns are specific to the pregnancy itself such as fears regarding the pregnancy, delivery, and health of the child [12].

### 2.2. Search Strategy

The three authors (Gianella Pana, M. Sarah Rose, and Shahirose Premji) independently searched the literature to retrieve potential studies that explored the relationship between prenatal maternal anxiety and PTB in two stages. Initially databases were searched using the exact search phrase: (prenatal OR antenatal OR pregnancy) AND (anxiety) AND (preterm OR premature OR prematurity); and the searches were limited to English, humans, and journal studies. All studies published up until June 2015 in MEDNINE (1946 to June 2015), Cumulative Index to Nursing and Allied Health Literature (1961 to June 2015), PsycINFO (1806 to June 2015), and EMBASE (1947 to June 2015) were extracted. The retrieved records were entered into Refworks and duplicates were removed. The titles of the studies were reviewed for obvious exclusion according to the study objective. Any SR or MA were separated from primary sources and screened for relevance. The abstracts of the remaining primary studies were then reviewed for relevance.

### 2.3. Types of Studies and Outcomes

Studies were considered relevant if they examined the relationship between any type of anxiety and PTB, measured either as a continuous (i.e., gestational age) or binary variable (PTB or spontaneous PTB).

### 2.4. Study Selection

Studies deemed to be appropriate were scanned in full to determine relevance. Secondly the references lists of all relevant studies were reviewed to find additional studies that may have been difficult to detect in the database search due to nonreporting in the abstract (possibly due to nonsignificant effects). Studies published by the same team were carefully reviewed to ensure the results of a given study were not included twice in the MA.

### 2.5. Data Extraction

Data was extracted independently by two reviewers (Gianella Pana and M. Sarah Rose) using a standardized review form and compared for discrepancies. Any discrepancies were discussed and agreement achieved. A standardized excel sheet was created and information from the standardized review forms was transferred in order to be readily available for the meta-analysis. The items extracted are presented in Table 1.

### 2.6. Critical Appraisal

The quality and validity of each study were assessed using the critical appraisal (CASP) tool [13] and the included studies were summarized in tabular form. The CASP questions are also included in Table 1.

In addition, a critical appraisal of the statistical methods used to analyze the data was done, and their relevance to the design and objectives of the study was assessed. We assessed methods used to develop multivariable models and adjusted estimates. In particular, we examined the methods used to include variables in the multivariable model (e.g., manual, forward stepwise, backward stepwise, and hierarchical). We assessed whether each included covariate was a potential confounding variable and whether it was highly correlated with the primary predictor variable. Two criteria for confounding are that the confounder must be associated with the outcome of interest and that the confounder must also be associated with the primary predictor variable. Collinearity occurs when two predictor variables in a regression model are so highly correlated that it becomes difficult or impossible to distinguish their individual effects on the outcome. Clearly a collinear variable qualifies as a confounding variable, but this is an extreme case of confounding when essentially the same variable is entered twice. Unfortunately, this may be a result of using self-report questionnaires where it may be impossible to determine participants that are depressed only, anxious only, both, or neither. Using a diagnostic tool may be able to do this but would be much more expensive to implement. Because of the inherent difficulty of interpreting the separate effects of highly correlated variables, the adjustment was considered appropriate if the variables in the model were potential confounding variables and not highly correlated with primary predictor variable. The questions for the critical appraisal are included in Table 1.

### 2.7. Potential Sources of Heterogeneity of Primary Interest

The primary sources of heterogeneity that we considered were as follows: (1) the primary predictor variable (i.e., type of anxiety, e.g., PSA, TA, SA, or anxiety disorder), (2) the primary outcome variable (gestational age, PTB, or spontaneous PTB), (3) the type of summary statistic (i.e., correlation coefficients (CC) or odds ratio (OR)), and (4) whether the estimate provided was unadjusted or adjusted and if adjusted whether this was considered an appropriate adjustment (see Section 2.6).

### 2.8. Statistical Methods

#### 2.8.1. Data Preparation

Studies that reported the results as a relative risk (RR) were converted to OR for consistency. In order to ensure that all measures were independent when one author contributed more than one estimate due to repeated measurements of anxiety, we used a single summary estimate providing that these estimates were homogenous. When a single study reported two estimates, one for African American Women and one for White Women, these OR were combined using a Mantel-Haenszel OR.

#### 2.8.2. Risk of Bias due to Confounding: Assessment and Management

Since all studies were observational in design, one of our primary concerns was the control of bias due to confounding. Some studies included only unadjusted estimates and some included only adjusted estimates. If there is substantial evidence of bias due to confounding then it would not make sense to include both unadjusted and adjusted estimates in the same meta-analysis. Consequently, we first assessed the extent of (potential) bias in the unadjusted estimates by examining the relationship between adjusted and unadjusted estimates (using a scatterplot and linear regression) including only studies that presented both estimates. We also examined the effect of inappropriate versus appropriate adjustment on the potential bias.

#### 2.8.3. Meta-Analysis

The relationship between prenatal maternal anxiety and PTB was summarized using one of two statistical estimates of effect sizes: (1) the OR when the primary outcome variable was PTB or spontaneous PTB (binary variables) or (2) the CC (transformed using Fisher's arc sine transformation) when the primary outcome variable was gestational age (continuous). The results are therefore reported separately for each of these two statistical estimates. Pooled estimates were based on fixed or random effects models depending on the degree of heterogeneity. Heterogeneity amongst the estimates was examined using the *Q* statistic (where *p* < 0.05 provides evidence against the assumption of homogeneity) and *I* ^2^ (which is the variation in the effect size due to heterogeneity). Results are illustrated using Forest Plots.

## 3. Results

**Figure 1:**
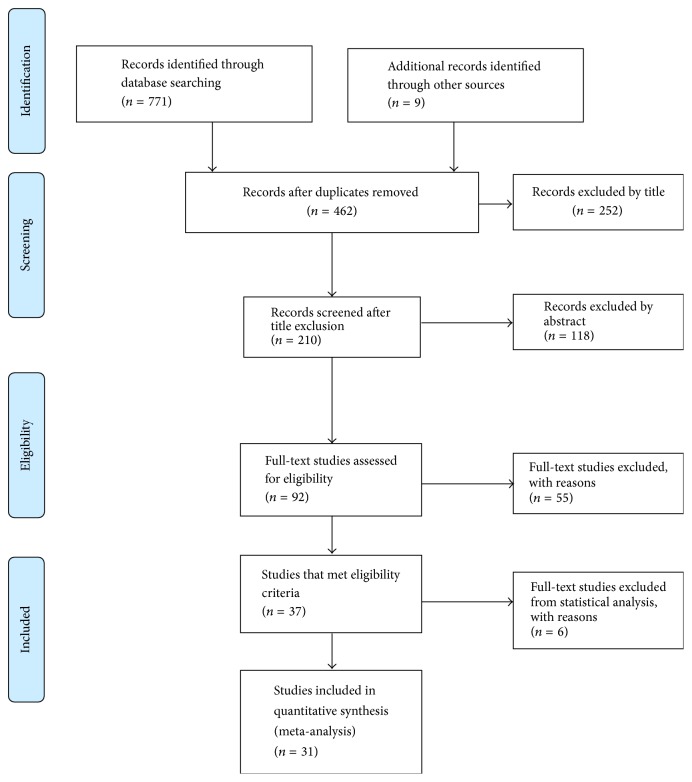
PRISMA flow diagram for inclusion of studies examining the relationship between prenatal anxiety and PTB.

A total of 780 studies were identified through database searching and reviewing reference lists with 462 studies remaining after duplicates were removed (Figure 1). After excluding by title (*N* = 252) and excluding by abstract (*N* = 118), a full-text review of 92 studies was conducted. From these 92 studies, 55 were excluded based on inclusion criteria leaving 37 studies, of which six [14–22] were excluded during data extraction since they did not provide enough information to calculate estimates, leaving 31 [23–50] studies eligible for the meta-analysis.

### 3.1. Critical Appraisal of Studies

Many of the 31 studies focused on the relationship between prenatal maternal anxiety and PTB (*N* = 30), had an unbiased measure of anxiety (*N* = 25), had an unbiased measure of gestational age and defined PTB (*N* = 26), accounted for identified confounders in their analysis (*N* = 22), and had a long enough follow-up of the subjects (*N* = 34). Many of these 31 studies, however, did not appear to have a cohort representative of the population (*N* = 18). Participants were usually recruited from hospital clinics, private practices, and walk-in clinics or were referred to the study by private practitioners. The method of sampling was not stated (e.g., sequential, systematic, random, or convenience) and participants were often selected as members of a particular subgroup (e.g., at risk of intrauterine growth restriction, low medical risk, high medical risk, low income, and availability of biomarker assays). In general, the consent rate was low, as was follow-up, so that the ratio of the size of the final sample compared to the size of the eligible sample was very low (as low as 33% in some studies). In addition, many studies did not identify all confounders (*N* = 21), and the design and methods of 18 of these studies were sufficiently flawed to make the results unreliable (Supplementary Table 1, see Supplementary Material available online at http://dx.doi.org/10.1155/2016/8312158). Overall, there were 18 studies that described the relationship between anxiety and PTB or spontaneous PTB using the OR (three studies provided two estimates) and 12 using the CC (six studies reported two estimates of the CC and five reported one only). One study provided information only in terms of the standardized mean difference and was therefore not included in the analysis. This resulted in 22 estimates of the OR and 17 estimates of the CC.

### 3.2. Data Management

Examination of the relationship between adjusted and unadjusted estimates of the OR in 11 estimates from eight studies indicated that there was no evidence against linearity of the relationship, deviation of the intercept from zero (estimated intercept = 0.03, 95% CI −0.06, 0.12, and *p* = 0.556 for difference from zero), or the slope from one (estimated slope = 0.93 (95% CI 0.79, 1.06), *p* = 0.272 for difference from unity). We, therefore, combined both unadjusted and adjusted estimates from the studies, and if a study provided both estimates, the adjusted estimate was used. For studies that used gestational age as the outcome variable, adjustment methods were too variable to consider combining adjusted estimates (i.e., structural equation modelling (*N* = 3), multiple linear regression (*N* = 5), and no adjusted estimate (*N* = 2)) so we focused on the CC only.

### 3.3. Meta-Analysis

**Figure 2:**
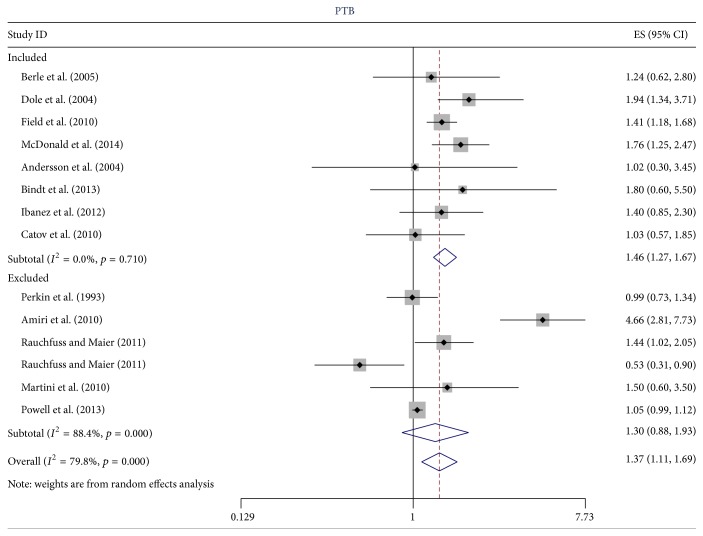
The effect of excluding estimates of dubious quality on the heterogeneity of the estimates of the OR for anxiety and PTB. There were three exclusion criteria: (1) the results of the study were numerically suspicious; (2) the authors reported the odds ratio for a continuous predictor variable; and (3) the odds ratio was inappropriately adjusted as described in Critical Appraisal.

**Figure 3:**
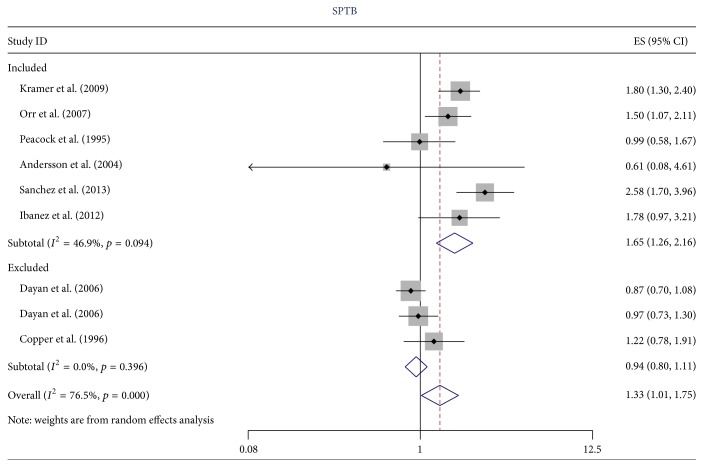
The effect of excluding estimates of dubious quality on the heterogeneity of the estimates of the OR for anxiety and spontaneous PTB. There were three exclusion criteria: (1) the results of the study were numerically suspicious; (2) the authors reported the odds ratio for a continuous predictor variable; and (3) the odds ratio was inappropriately adjusted as described in Critical Appraisal.

We initially categorised the studies into five groups according to the outcome variable and the type of analysis, as illustrated in Table 2: OR for spontaneous PTB (*N* = 9); OR for PTB (*N* = 13); correlation for PTB (*N* = 3); and correlation with gestational age (*N* = 10). These numbers do not total 31 since some studies reported more than one estimate and we had to exclude one study since we were unable to extract information [19]. There was substantial heterogeneity across the studies for those reporting OR for spontaneous PTB (*I* ^2^ = 76.0%, *p* < 0.001) and PTB (*I* ^2^ = 79.8%, *p* < 0.001). When studies that used inappropriate methods of adjustment [19–22] or reported the OR for a unit or 5-unit increase in anxiety [29, 47, 51] were removed the heterogeneity was substantially reduced (*I* ^2^ = 46.9%, *p* = 0.094 for spontaneous PTB and *I* ^2^ = 0.0%, *p* = 0.710 for PTB). In Figure 2 (PTB) and Figure 3 (spontaneous PTB) we illustrate the reduction in heterogeneity in excluding these studies. There was little or no evidence of heterogeneity for the three studies reporting the CC when the outcome was PTB (*I* ^2^ = 61.9%, *p* = 0.073) and for the 13 studies that used gestational age as the outcome variable (*I* ^2^ = 0.0, *p* = 0.570).

**Figure 4:**
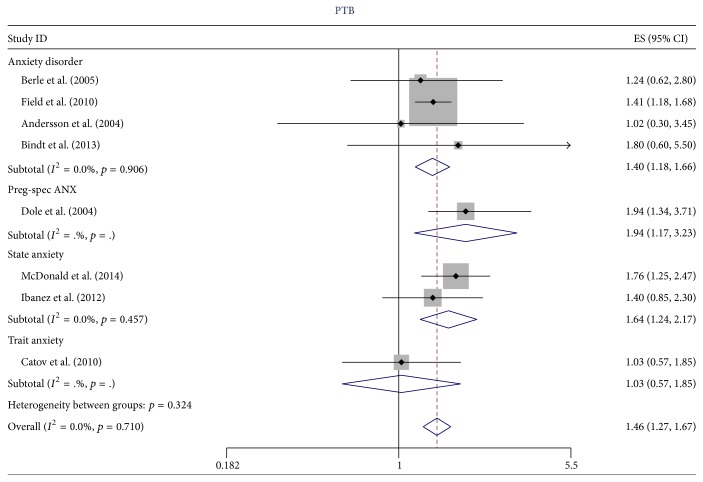
The effect of type of anxiety on the estimate of the odds ratio for the relationship between anxiety and PTB.

**Figure 5:**
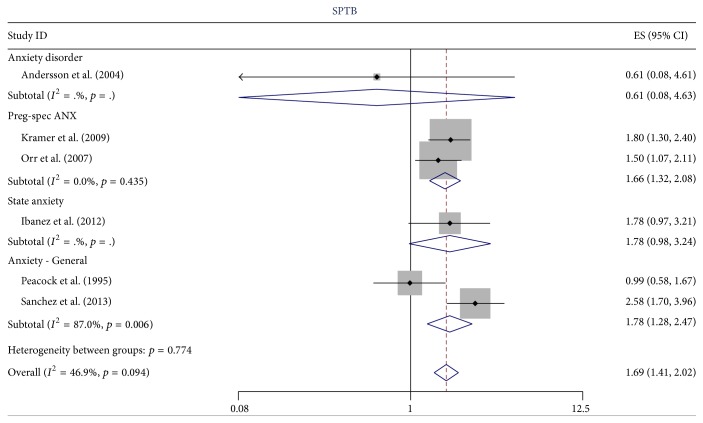
The effect of type of anxiety on the estimate of the odds ratio for the relationship between anxiety and spontaneous PTB.

**Figure 6:**
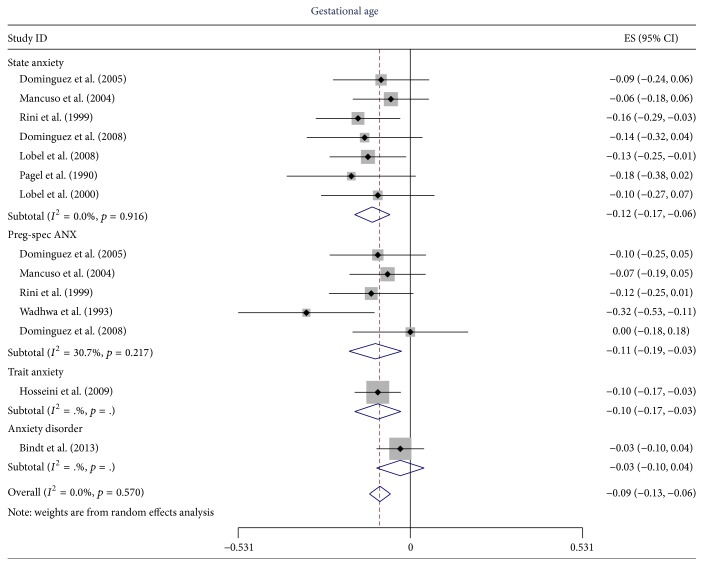
The effect of type of anxiety on the estimate of the correlation coefficient (ES) between anxiety (measured as a continuous variable) and gestational age.

For the 24 studies that remained after these exclusions, five studies used anxiety disorder for the primary predictor variable, eight used PSA, 10 used SA, two used TA, and two used gestational age (note some used more than one). Since there was no evidence of heterogeneity for the OR for PTB, we combined all types of anxiety measured (anxiety disorder (*N* = 4), SA (*N* = 2), and PSA (*N* = 1)) for an overall summary OR of 1.46 (95% CI 1.27, 1.67), as illustrated in Figure 4. When the primary predictor variable was restricted to SA and PSA the estimate was (OR = 1.70, 95% CI 1.33, 2.18, *N* = 3) for PTB. For spontaneous PTB, the summary OR for all types of anxiety was 1.69 (95% CI 1.41, 2.02) as illustrated in Figure 5, but when heterogeneity was reduced (*I* ^2^ = 0.0%, *p* = 0.774) by using only estimates of SA and PSA the summary OR was almost identical 1.67 but the 95% CI was wider (95% CI 1.35, 2.07). The summary CC were almost identical −0.09 (95% CI −0.13, −0.06) for gestational age and −0.09 (95% CI −0.12, −0.06) for PTB. When restricted to SA and PSA for gestational age the CC were −0.12 (95% CI −0.17, −0.06) and −0.11 (95% CI −0.19, −0.03), respectively, as illustrated in Figure 6. These were not combined since five of the authors contributed estimates to both.

## 4. Discussion

### 4.1. Summary

We found the most precise estimates of the relationship between prenatal maternal anxiety and PTB when we restricted our analysis to SA (OR = 1.70 (95% CI 1.33, 2.18) for PTB, *N* = 3) and PSA (OR = 1.67, (95% CI 1.35, 2.07) for PTB, *N* = 3). When gestational age was the outcome variable the summary CC was −0.12 (95% CI −0.17, −0.06) for SA and −0.11 (95% CI −0.19, −0.03) for PSA. We did not combine these estimates since 4 of the studies included estimates for both SA and PSA. The estimates of increased risk of PTB are almost identical for both SA and PSA. This is not surprising since these variables have been found to be very highly correlated in both validation studies [52] and studies in this review [10, 32, 33, 40, 41, 48]. There could be several reasons for this: (1) it may not be possible to separate SA and PSA using self-report questionnaires, (2) both types of anxiety have the same physiological response which may lead to PTB, and (3) SA may be a natural sequelae of PSA or vice versa. Studies suggest that PSA or fear of childbirth is more prevalent among women with high SA [53–55]. SA relates to the temporary or emotional anxiety aroused by a situation or circumstance and is assessed using a 20-item Spielberger State and Trait Anxiety Inventory Form Y-1 [56, 57]. PSA, on the other hand, can be assessed with a 10-item Pregnancy-Related Anxiety Scale-revised [48] and unlike the Spielberger State and Trait Anxiety Inventory Form Y-1 has no cost attached to it; thus, is cost-effective when considering implementation of a screening program.

### 4.2. Comparisons with Other SRMA

There have been five reviews which have, in part, examined the relationship between prenatal maternal anxiety and PTB [3, 7, 9, 10, 58]. Three of these have been narrative [3, 7, 58], whereas two have produced summary statistics from a MA [9, 10]. Dunkel-Schetter and Glynn [3] provided a narrative review which was the most comprehensive in that her bibliography included 21/23 papers in our review published prior to 2010. They separated anxiety into anxiety (general; *N* = 11) and PSA (*N* = 9) and one situational anxiety [18]. Their conclusions were vague; “a total of 6 of the 11 studies on general or state anxiety show some impact on preterm birth or gestational age, although in all cases the effects are somehow qualified” [3]. They also indicated that all of the eight studies, which examined PSA, showed an effect on PTB.

Alder et al. [58] provided a selective narrative review, in which only 5/17 studies we found prior to 2007 were included in her bibliography. Only two of these, however, were discussed in the section of the effect of maternal anxiety and depression on gestational age, from which they concluded that there was no relation to gestational age with enhanced levels of anxiety. The final narrative review [7] was selective with only 13/29 studies published prior to 2012 included. The authors concluded that anxiety (and general perceptions of stress) has been associated with shortened gestation in many (*N* = 9/11) studies.

Ding et al. [9] included 12/31 studies that we found prior to 2013 in their analysis, but they purposefully omitted studies that did not include an OR; eight of these we included in the current MA, but we excluded four due to problems with the reported statistical analysis. We also included another four studies, which were published after Ding et al.'s [9] MA was published. Ding et al. [9] found that prenatal maternal anxiety was significantly associated with an increased risk for PTB, but their summary relative risk (RR = 1.5 (95% CI 1.33, 1.70)) included 12 studies which had a mixture of outcome (PTB and spontaneous PTB) and types of anxiety (SA, TA, anxiety disorder, and PSA) and included both adjusted and unadjusted estimates. Surprisingly they found no evidence of heterogeneity amongst these 12 studies, whereas we found substantial evidence of heterogeneity. Littleton et al. [10], on the other hand, provided a MA for studies that reported CC. They identified five of the studies that we found and provided a mean CC of −0.06 (95% CI −0.11 to −0.02) for 10 studies, but despite the 95% CI not including zero, they claimed that there were no associations between anxiety symptoms and perinatal outcomes, due to their “Fail-safe” *p*-values. This method has been criticised and the Cochrane handbook recommends that these methods not be used [14]. They also found a mean CC of −0.10 (95% CI −0.24, −0.06) for five studies that looked at the relationship between PSA and gestational age.

### 4.3. Strengths and Limitations of Our Meta-Analysis

Unlike previous narrative reviews [3, 7] and meta-analysis [9], we did not find any evidence to suggest that PSA has a greater risk than SA, although the number of studies was small but consistent. While one meta-analysis [10] found a small relationship between PSA and PTB the estimates were below established fail state cut-off; thus, the reliability of the findings was questioned. Unlike previous meta-analysis, we separated studies which used the CC from those that used an OR, since these are inherently different statistics. We decided to investigate the summary OR and CC separately although methods exist to convert both these measures to an effect size [59]. We did this primarily because of the potential inappropriateness of the CC, which is difficult to determine without access to the individual level data. Whether the relationship between a measure of anxiety and gestational age is linear is doubtful, which increases the difficulty of interpreting CC in this context. Another point to bear in mind is that since neither variable is known to be normally distributed the CC is in general not a good measure of the strength of the relationship. However, both the summary OR and the summary CC do have equivalent effect sizes. An OR of 1.7 with a 95% CI 1.3, 2.1 is equivalent to a CC of −0.11 with a 95% CI −0.16, −0.06. So we can conclude that our two analyses are consistent.

### 4.4. Heterogeneity in Meta-Analyses

Thompson makes a distinction between statistical and clinical heterogeneity [60]. Clinical heterogeneity arises when the included studies differ in terms of patient selection and methodological differences, such as study design and differently defined primary predictor and outcome variables. Statistical heterogeneity, as determined by a significant *Q* statistic or *I* ^2^, may be caused by these known clinical and methodological differences or it may be caused by unknown or unrecorded clinical and methodological differences. Thompson, among many other authors, emphasizes that sources of heterogeneity must be investigated to increase the clinical relevance of the conclusions [60].

We reduced statistical heterogeneity substantially by omitting studies that were apparently numerically incorrect or had inappropriately adjusted estimates of the OR (e.g., that included another highly correlated predictor variable such as another type of anxiety or depression). In addition we did not combine estimates that did not make sense to combine, such as an OR when the predictor variable is binary with one in which the predictor variable is continuous. Although it is possible to use a random effects model to estimate the summary OR in the presence of statistical heterogeneity Thompson [60] points out that this is only useful if the statistical heterogeneity cannot be explained by clinical differences. In our analysis we focused on methodological differences particularly in terms of the operationalization of the primary variables and the statistical methodology.

### 4.5. Issues in Statistical Methodology

If we consider the situation when PTB is a dichotomous variable and anxiety as a continuous variable, it is important that the assumption of linearity between the log-odds of PTB and anxiety score is not violated. Interpretation of the OR per k-unit increase is difficult since this model assumes that the OR is the same when comparing a score of 20 with a score of 15, both of which are very low and when comparing a score of 42 with a score of 47, when both scores are around the cut-off level for high anxiety. Combining an OR expressed as a unit or 5 unit increase in the primary predictor variable with an OR when the primary predictor variable is binary is not appropriate because even within the same data these estimates will be different. Consider an example of a simulated dataset in which the CC between gestational age and anxiety is −0.2, and the variables are similarly distributed as those in the studies included in our SR and MA. The predictor variable is a measure initially on a continuous scale such as the STAI but then may be dichotomised using, for example, the 75th percentile. We could report the OR per unit or per 5-unit increase in the continuous anxiety score (e.g., in our example the coefficients would be 1.04 per unit increase or 1.25 per 5-unit increase). However, if we choose to dichotomise the predictor variable the OR will be quite different, since it is comparing one group with a range of scores to another with a different range of scores. In our example the OR for the binary predictor is 2.2.

We had to exclude four adjusted estimates from the MA [19–22]. In these studies the authors had included anxiety as a continuous variable in the model along with a highly correlated predictor variable. Examples of highly correlated predictor variables are two measures of the same anxiety scale taken at different times during pregnancy; two different measures of anxiety (e.g., SA and PSA), and depression and anxiety. In each case the correlation between the variables is very high (in the order of 0.5 to 0.7) which will result in collinearity and difficulty in interpreting the resulting coefficients.

### 4.6. Limitations

#### 4.6.1. Ethnicity

We would have liked to assess the effect of ethnicity on the relationship between anxiety and PTB, but it is not impossible for us to assess in the present study. In many of the North American studies the populations were a mix of Caucasian, Black, Hispanic, and Other ethnicities some of which were not even specified. We have now included Table 3 in the paper, which includes the percentage of White, Black, and Hispanic women in the sample for each study. Most papers did not address the issue of ethnicity and indeed would not have been powered to do so. There was only one paper in our MA that produced separate estimates of the OR for Black and White women separately. Dole et al. [31] found an OR of 2.2 (95% CI 1.3 to 3.7) for Black women and 1.7 (95% CI 1.1, 2.5) for White; thus, there was a large overlap in the 95% CI of these estimates in spite of the large sample sizes (*N* = 644 Black women and 1098 White women). We therefore decided to combine the estimates, using the inverse variance method resulting in an estimate of 1.94 (1.34, 2.82). The estimate of *I* ^2^ was 0.0% and *p* = 0.430 for the *Q* statistic. We felt that it was more appropriate to combine these estimates rather than to treat them as two separate studies in the meta-analysis. Catov et al. [28] presented estimated for Black and White women, but since this analysis was of subset of data from a previous analysis [27] we chose not to duplicate this in our MA. Interestingly in this subsequent analysis, there was no effect of anxiety of PTB in either Black or White women and the ORs were very similar: 1.4 (95% CI 0.34, 5.0) for Black women and 1.6 (95% CI 0.6, 3.7) for White women.

#### 4.6.2. Exposure

We had originally planned to examine the effect of exposure (degree of anxiety) but this became impossible to do. There were more than thirteen validated scales used amongst the 37 studies, and many of these had been adapted in some form. Additionally many scales were “Researcher Developed” for the purpose of the study and others adapted from some Researcher Developed scales. Even for the validated scales, different cut-off values were used and some were intrinsically binary (such as diagnoses). We have included these in Table 3, which may help describe the sources of heterogeneity across the studies.

#### 4.6.3. Other Sources of Bias

Several limitations of this MA arise from the inherent limitations of the included studies as evident in the critical appraisal conducted. Many studies did not have a cohort representative of the population, as described earlier, making it difficult to generalize the results to all pregnant women with or without anxiety. Many of the included studies did not identify and account for all confounding variables, making it difficult to determine if their results are valid, whereas other studies inappropriately adjusted their estimates with variable highly correlated with anxiety. In addition, eight of the 35 studies that looked at the relationship between anxiety and PTB were excluded because of the limited information they provided. We did not examine publication bias using Funnel plots since it has been determined that this analysis has very little power, especially with a small number of studies [61, 62].

### 4.7. Future Directions

Our investigation shows consistent findings that there is a statistically significant association between maternal anxiety during pregnancy and PTB; the results, however, cannot assume causality. The relationship between maternal anxiety during pregnancy and PTB currently satisfies the Bradford Hill criteria [63] of specificity (pregnant women giving birth to preterm babies), temporality (prenatal anxiety occurs before PTB), and, with the addition of the results of this paper, consistency. In order to further satisfy the Bradford Hill criteria it would be practical to create a prenatal program designed to reduce PSA and/or SA in pregnant women. This intervention could then be utilized in a randomized-control trial (RCT) to determine if the reduction in PSA improves PTB rates. If the results of the RCT show that there is a statistically significant difference between the control group and the group with the anxiety reduction intervention, then we can begin to assume that PSA causes PTB and this would have enormous implications for health promotion in pregnant women. We identified that the OR for prenatal anxiety and PTB is of the order of 1.3 to 2.0 (considering the limits of the CI). What does this mean on a global level? We used data provided by Blencowe et al. [64] which reports the number of births and the preterm birth rate in seven different regions of the world in 2010. If we estimate that 25% of pregnant women have some form of anxiety and take a very conservative estimate of the RR of about 1.3, then in Northern Africa and Western Asia, the number of PTB that could be prevented by treating anxiety during pregnancy (Attributable Fraction) would be very close to 44,000 and in Southern Asia would be 303,000 in one year. If we consider a less conservative RR (2.0) the number prevented in Northern Africa and Western Asia would be 150,000 and over 1,000,000 in Southern Asia.

## 5. Conclusions

There was substantial heterogeneity across the studies for those reporting OR for spontaneous PTB and PTB, but after excluding studies that used inappropriate methods of adjustment or reported the OR for a unit or 5-unit increase in anxiety, the heterogeneity was substantially reduced. Further reductions in heterogeneity were observed when the primary predictor variable was restricted to SA and PSA. Consistent findings indicate a significant association between prenatal maternal anxiety and PTB; therefore, a prenatal program designed to reduce maternal anxiety during pregnancy could decrease the burden of PTB on the healthcare system.

## Supplementary Material

The Table in the supplementary material presents the details of the critical appraisal according to the Critical Appraisal Skills Program (CASP) tool in which the quality and validity of each study is assessed. The nine CASP questions addressing the potential for bias are provided in Table 1 of the manuscript and each is answered with a Yes, Can't tell or No.

## Figures and Tables

**Table 1:** Items on the structured data extraction form, the CASP tool for CRA, and the appraisal of the statistical analysis.

Methods	Results
First author	Age
Year of publication	Education
Other authors	SES or Poverty Index
Country	Marital status
Location	Smoking
Journal	Alcohol problem
Data collection dates	
Key words	*Primary outcome*
Type of study	Gestational age (days)
Number of and time points for observation	Preterm birth (<259 days or <37 w)
Inclusion/exclusion	
Existing study name	*Primary predictor variable*
Sample size	Descriptive analysis
Consent rate, participation rate	*Relationships*
Primary predictor variable	Unadjusted relationships
Measurement of PV	Adjusted relationships
Other predictor variables	
Outcome	Additional comments
Potential confounders	

CASP	Statistical analysis

Is the clearly focused issue relevant to our study (anxiety and preterm birth)?	Unadjusted analysis:statistic and test
Was the cohort recruited in an acceptable way? That is, is the cohort representative of the population it is supposed to represent?	Appropriate? Numerically correct?
Was the outcome (preterm birth) accurately measured to minimise bias?	Method of adjustment; type of model Details of model development
Have the authors identified all-important confounders? (Age, marital status, ethnicity, education, income or SES, parity, previous PTB)	Appropriate confounders considered?
And have they accounted for this in the analysis?	Appropriate control of confounding? Assessment of linearity assumption
Follow-up: completeness	Methods for missing data specified
Follow-up: length (note generally not a concern in pregnancy studies)	Overall quality of adjusted analysis
Do you believe the results? (on a scientific basis and gut feeling)	Other comments

**(a):** 

Author	Year	Inclusion	Country	Study design	Ethnicity				Statistic	Outcome
					Ethnicity	W	H	B		
Berle et al. [25]	2005	0	Norway	CS	Norway				OR	PTB

Copper et al. [29]	1996	2	USA	PC	B, W, H	35%	1%	63%	OR	SPTB (<35 w)

Dole et al. [31]	2004	0	USA	PC	B, W	62%		38%	OR	PTB: SPTB

Dominguez et al. [33]	2005	0	USA	PC	B			100%	CC	GA

Field et al. [34]	2010	0	USA	PC	B, W, H	9%	59%	32%	OR	PTB

Glynn et al. [35]	2008	0	USA	PC	B, W, H	48%	23%	14%	OR	PTB

Goldenberg et al. [19]	1996	1	USA	PC	B, W	31%	69%			PTB

Hosseini et al. [36]	2009	0	USA	PC	B, W	49%		51%	CC	GA

Kramer et al. [38]	2009	0	Canada	PC	B, W, H	80%	5%	8%	OR	SPTB

Lobel et al. [41]	2000	0	USA	PC	W	87%			CC	GA

Mancuso et al. [42]	2004	0	USA	PC	B, W, H	24%	32%	43%	CC	GA

McDonald et al. [43]	2014	0	Canada	PC	W	80%			OR	PTB

Orr et al. [44]	2007	0	USA	PC	B, W	23%		77%	OR	SPTB

Peacock et al. [46]	1995	0	England	PC	W	100%			OR	SPTB

Perkin et al. [20]	1993	2	England	PC	W	100%			OR	PTB

Rini et al. [48]	1999	0	USA	PC	W, H	48%	52%		CC	GA

Roesch et al. [14]	2004	1	USA	PC	B, W, H	23%	35%	43%		PTB: GA

Uguz et al. [15]	2013	1	Turkey	CS	Turkey					GA

Wadhwa et al. [50]	1993	0	USA	PC	B, W, H	77%	13%	7%	OR, CC	PTB: GA

Bhagwanani et al. [16]	1997	1	USA	PC	B, W, H	65%	8%	27%		PTB

Andersson et al. [24]	2004	0	Sweden	PC	Sweden				OR	PTB: SPTB

Bindt et al. [26]	2013	0	G/C D'I^*∗*^	PC	G/C D'I^*∗*^				OR, CC	PTB: GA

Bödecs et al. [17]	2011	1	Hungary	PC	Hungary					PTB

Catov et al. [27]	2010	0	USA	PC	B, W	70%		30.0%	OR	PTB

Dayan et al. [30]	2006	2	France	PC	W	94%			OR	PSTB

Dominguez et al. [32]	2008	0	USA	PC	B, W	100%		100%	CC	GA

Latendresse and Ruiz [39]	2011	0	USA	PC	B, W, H	69%	23%	4%		PTB

Lobel et al. [40]	2008	0	USA	PC	B, W, H	65%	12%	12%	OR	SPTB: GA

Amiri et al. [23]	2010	2	Iran	PC	Iran				OR	PTB

Rauchfuss and Maier [21]	2011	2	Germany	PC	Germany				OR	PTB

Sanchez et al. [49]	2013	0	Peru	CC	Peru				OR	SPTB

Martini et al. [22]	2010	2		PC	Germany				OR	PTB

Powell et al. [47]	2013	2	Australia	RCT	Australia				OR	PTB

Ibanez et al. [37]	2012	0	France	PC	France				OR	PTB: SPTB

Levi et al. [18]	1989	1	Sweden	PC	Sweden					PTB: GA

Pagel et al. [45]	1990	0	USA	PC	B, W	78%		7%	C	GA

^*∗*^Ghana/Cote D'Ivoire.

**(b):** 

Author	Year	PPV	Scale	# Items	Item score score	Range	Cut-off	# Obs	Times	MMM	Trimester
Berle et al. [25]	2005	Anxiety	HADS-A^*∗*^	7	1_4	0–21	≥8	1	Anytime		

Copper et al. [29]	1996	Trait	STAI^*∗*^	5	1_5	20	C	1	26 ± 0.8 w		L2/E3

Dole et al. [31]	2004	PSA	PSIS^*∗*^	6	0_3	18		1	24–29 w		L2/E3

Dominguez et al. [33]	2005	State, PSA	STAI, RD	10	1_4	10–40	ns	3	18–20 w, 24–26 w, 32–36 w	Mean	2/3

Field et al. [34]	2010	Anxiety	SCID	20		20–90	48	1	20 w		2

Glynn et al. [35]	2008	PSA	Rini^*∗*^	10	1_4	10–40		2	19.3 w, 31.0 w	Both	2/E3

Goldenberg et al. [19]	1996	Trait	STAI	ns	ns	ns	ns	1	24–26 w		L2

Hosseini et al. [36]	2009	Trait	STPI	10	1_4	10–40	NA	2	4 m, 7 m	First	2

Kramer et al. [38]	2009	PSA	D-S^*∗*^	4	1_5	16	Q	1	24–26 w		L2

Lobel et al. [41]	2000	State	STAI	20	1_4	60		3	10–20 w, 21–30 w, >31 w	3 CC	1/E3

Mancuso et al. [42]	2004	PSA: State	RD; STAI	4	1_5	16	NA	3	18–20 w, 28–30 w	2 CC	2/E3

McDonald et al. [43]	2014	State	STAI	20	1_5		40	1	<25 w		L2

Orr et al. [44]	2007	PSA	PSEI^*∗*^	6	0_1	0–6	≥4	1	1st prenatal visit		1

Peacock et al. [46]	1995	Anxiety	GHQ			0–21	Q	1	b		1

Perkin et al. [20]	1993	Anxiety	GHQ				Q	3	b, 28 w	Max	1/E3

Rini et al. [48]	1999	PSA	Wadhwa^*∗*^	10	1_4	30		1	28–32 w		E3

Roesch et al. [14]	2004	State	STAI	10	1_4	30		3	18 w, 28 w		L/E3

Uguz et al. [15]	2013	PSA	PSA	4	1_5	16		1	36 w GA - 8 w PP	First	3

Wadhwa et al. [50]	1993	PSA	PAIP^*∗*^	5	0_1	0–5	C	1	28–30 w		L2/E3

Bhagwanani et al. [16]	1997	PSA	RD	5	1_5	20		5	8–28 w, then + 6 w		1/E3

Andersson et al. [24]	2004	AD	PRIME-MD				B	1	16–18 w		2

Bindt et al. [26]	2013	AD	GAD-7	7	0_3	0–21	≥10	1	3 trimester		3

Bödecs et al. [17]	2011	AD	GAD-7					1	M = 8.13		1

Catov et al. [27]	2010	Trait	STAI-T	10	1_4	10–40	>20	1	M = 17.9		L2/E3

Dayan et al. [30]	2006	State: Trait	STAI-Y	40	1_4	20–80	C	1	20–28 w		2

Dominguez et al. [32]	2008	State: Trait	STAI-Y	40	1_4	60		3	18–20 w, 24–26 w, 30–32 w	Mean	2/E3

Latendresse and Ruiz [39]	2011	PSA	RD		1_5			1	14–20		2

Lobel et al. [40]	2008	State	STAI					3	10–25 w, 21–30 w, >30 w	3 CC	L1/2

Amiri et al. [23]	2010	State	STPI	10	1_4	30		1	20–28 w		L2/E3

Rauchfuss and Maier [21]	2011	Anxiety: PSA	RD; Lukesch^*∗*^	5,3		0–5 0–6		1	13–24 w		2

Sanchez et al. [49]	2013	Anxiety	DASS-21	4			≥10	1	PP		PP

Martini et al. [22]	2010	AD	DSM-IV				B	1	PP		PP

Powell et al. [47]	2013	State: Trait	STAI-6				C	1	Mean 19.7		L2/E3

Ibanez et al. [37]	2012	State	STAI	20	1_4	20–80	≥37	1	24–28 w		L2/E3

Levi et al. [18]	1989	S, P, C	CAI	7	?	?	?	1	36 w		3

Pagel et al. [45]	1990	State	STAI	20	1_4	20–80		1	21-36 w		L2/E3

**(c):** 

A: inclusion	*Whether the paper identified the systematic review was included in the meta-analysis*	*0*	*Yes*
		*1*	*Excluded in the first stage*
		*2*	*Excluded in the second stage*

A: study design		CS	Cross-sectional
		PC	Prospective cohort
		RCT	Randomised controlled trials

A: ethnicity	The percentages of White, Black, and Hispanic women in the sample are given where available	B	Black
		W	White
		H	Hispanic
	Where no information was given on ethnicity, the country in which the participants were recruited is given. Note that the percentages do not always add to 100%, this is due to different “other” categories		

A: statistic	Whether the data were summarized using an OR or CC or both	OR	Odds ratio
		CC	Correlation coefficient

A: outcome	Whether the outcome was measured as gestational age (GA) in weeks or as a binary PTB or SPTB	PTB SPTB	(Spontaneous) preterm birth < 37 weeks (w); GA unless otherwise stated Spontaneous preterm birth < 37 weeks GA unless otherwise stated

B: PPV	The primary predictor variable (i.e., the type of anxiety)	Anxiety	General or not otherwise specified
		Trait	Trait anxiety
		State	State anxiety
		PSA	Pregnancy specific anxiety
		AD	Anxiety disorder

B: scale	*The scale used to measure the type of anxiety* ^*∗*^Indicates adapted (e.g., some items were omitted such as somatic complaints) Wadhwa^*∗*^, Rini^*∗*^, Dunkell-Schetter^*∗*^, Lukesch^*∗*^ all refer to PSA scales adapted from those designed by these original authors	HADS –A	Hospital anxiety and depression rating scale, anxiety
		STPI	State-trait personality inventory
		STAI (-T, -Y, -6)	The Spielberger sate and trait anxiety scale and various versions of this
		PSIS	Prenatal social inventory scale (Orr)
		PSEI	Prenatal social environment inventory
		GHQ	General health questionnaire
		PAIP	Psychosocial adaptation in pregnancy
		GAD-7	Generalized anxiety disorder
		DASS-21	Depression and anxiety stress scale
		CAI	Chernobyl anxiety index
		PRIME - MD	Primary care evaluation of mental disorders
		RD	Researcher developed
		SCID	Structured clinical interview of DSM-IV disorders

B: # items	The number of items in the scale		

B: scale	The scoring for each item		1_4 indicates 1, 2, 3, or 4

B: range	Where possible the limits of the range are given, for example, 20–80. Otherwise the width of the range is given		

B: cut-off	The cut-point at which the scales was divided to indicate low compared with high anxiety	*N*	Not clear whether the cut-point is > or ≥*N*
		≥*N*	
		C	The scale was used in as a continuous variable and no cut-off was used
		Q	The quartiles from the sample were used to define cut-points but were not always specified
		B	The anxiety disorder has been determined by diagnosis

B: # Obs.	Number of observations indicate the number of times that the PPV was measured during pregnancy		

B: times	The times during pregnancy that the measurement was taken		Booking (b); weeks (w)

B: MMM	The method of the author used to deal with the multiple measurements	First	The first measurement was used
		Mean	The mean of all 3 scores was taken
		Both	Both variables were included simultaneously in the model
		Max	Maximum value at any point during a particular woman's pregnancy
		3 CC	Three correlation coefficients were calculated

B: trimester	The trimester(s) in which most of the measures in each study were probably taken Derived from the times	1	First
		2	Second
		3	Third
		E	Early
		L	Late

**Table 3:** Classification of studies according to whether the primary outcome variable was PTB, spontaneous PTB, or gestational age and the type of statistic (odds ratio or correlation coefficient) used to estimate the relationship (top panel). The effect of excluding estimates of dubious quality on the heterogeneity of the estimates of the OR for anxiety and spontaneous PTB. There were three exclusion criteria: (1) the results of the study were numerically suspect; (2) the authors' reported the odds ratio for a continuous predictor variable; (3) the odds ratio was inappropriately adjusted as described in Section 2.6 (lower panel).

Statistic	Odds ratio		Correlation coefficient	
Outcome	SPTB	PTB	PTB	GA

Study	Andersson et al. (2004) [24] Dole et al. (2004) [31] Kramer et al. (2009) [38] Ibanez et al. (2012) [37] Peacock et al. (1995) [46] Sanchez et al. (2013) [49] Orr et al. (2007)^§^ [44] Copper et al. (1996)^*∗*^ [29] Dayan et al. (2006)^*∗*^ [30]	Berle et al. (2005) [25] Field et al. (2010) [34] Andersson et al. (2004) [24] Bindt et al. (2013) [26] Dole et al. (2004) [41] McDonald et al. (2014) [43] Ibanez et al. (2012) [37] Amiri et al. (2010)^*∗∗∗*^ [23] Catov et al. (2010) [27] Powell et al. (2013)^*∗*^ [47] Perkin et al. (1993)^*∗∗*^ [20] Martini et al. (2010)^*∗∗*^ [22] Rauchfuss and Maier (2011)^*∗∗*^ [21]	Glynn et al. (2008) [35] Lobel et al. (2008) [40] Wadhwa et al. (1993) [50]	Bindt et al.(2011) [26] Wadhwa et al. (1993) [50] Dominguez et al. (2005) [33] Dominguez et al. (2008) [32] Mancuso et al. (2004) [42] Rini et al. (1999) [48] Lobel et al. (2008) [40] Pagel et al. (1990) [45] Lobel et al. (2000) [41] Hosseini et al. (2009) [36]

*Heterogeneity*				
Before exclusion	76.0%, *p* < 0.001	79.8%, *p* < 0.001	61.9%, *p* = 0.073	0.0, *p* = 0.570
After exclusion	46.9%, *p* = 0.094	0.0%, *p* = 0.715		

Goldenberg et al. [19] study (1996) was excluded since it was not possible to extract any relevant information and Latendresse and Ruiz [39] (2011) only provided information on the mean (SD) anxiety scores in the mothers of preterm and those of term babies.

^§^Studies that provided adjusted estimates but not in the same form as the unadjusted estimate (i.e., for categorical rather than binary) so the unadjusted estimate was used.

^*∗*^Studies that reported the OR for a continuous predictor variable (excluded).

^*∗∗*^Studies that used inappropriate adjustment in the multivariable analysis and no unadjusted estimate available (excluded).

^*∗∗∗*^Studies that were numerically suspect (excluded).

## References

[1] Wen S. W., Smith G., Yang Q., Walker M. (2004). Epidemiology of preterm birth and neonatal outcome. *Seminars in Fetal and Neonatal Medicine*.

[2] Goldenberg R. L., Culhane J. F., Iams J. D., Romero R. (2008). Epidemiology and causes of preterm birth. *The Lancet*.

[3] Dunkel-Schetter C., Glynn L. M. (2011). Stress in pregnancy: empirical evidence and theoretical issues to guide interdisciplinary research. *The Handbook of Stress Science: Biology, Psychology and Health*.

[4] Bruce L., Beland D., Bowen A. (2012). MotherFirst: developing a maternal mental health strategy in Saskatchewan. *Healthcare Policy*.

[5] Ruiz R. J., Fullerton J., Dudley D. J. (2003). The interrelationship of maternal stress, endocrine factors and inflammation on gestational length. *Obstetrical and Gynecological Survey*.

[6] Wadhwa P. D., Culhane J. F., Rauh V., Barve S. S. (2001). Stress and preterm birth: neuroendocrine, immune/inflammatory, and vascular mechanisms. *Maternal and Child Health Journal*.

[7] Shapiro G. D., Fraser W. D., Frasch M. G., Séguin J. R. (2013). Psychosocial stress in pregnancy and preterm birth: associations and mechanisms. *Journal of Perinatal Medicine*.

[8] Uman L. S. (2011). Systematic reviews and meta-analyses. *Journal of the Canadian Academy of Child and Adolescent Psychiatry*.

[9] Ding X.-X., Wu Y.-L., Xu S.-J. (2014). Maternal anxiety during pregnancy and adverse birth outcomes: a systematic review and meta-analysis of prospective cohort studies. *Journal of Affective Disorders*.

[10] Littleton H. L., Breitkopf C. R., Berenson A. B. (2007). Correlates of anxiety symptoms during pregnancy and association with perinatal outcomes: a meta-analysis. *American Journal of Obstetrics and Gynecology*.

[11] Zeidner M. (2010). Anxiety. *International Encyclopedia of Education*.

[12] Blair M. M., Glynn L. M., Sandman C. A., Davis E. P. (2011). Prenatal maternal anxiety and early childhood temperament. *Stress*.

[13] CASP Checklist http://www.casp-uk.net/#!checklists/cb36.

[14] Roesch S. C., Schetter C. D., Woo G., Hobel C. J. (2004). Modeling the types and timing of stress in pregnancy. *Anxiety, Stress and Coping*.

[15] Uguz F., Sahingoz M., Sonmez E. O. (2013). The effects of maternal major depression, generalized anxiety disorder, and panic disorder on birth weight and gestational age: a comparative study. *Journal of Psychosomatic Research*.

[16] Bhagwanani S. G., Seagraves K., Dierker L. J., Lax M. (1997). Relationship between prenatal anxiety and perinatal outcome in nulliparous women: a prospective study. *Journal of the National Medical Association*.

[17] Bödecs T., Horváth B., Szilágyi E., Gonda X., Rihmer Z., Sándor J. (2011). Effects of depression, anxiety, self-esteem, and health behaviour on neonatal outcomes in a population-based Hungarian sample. *European Journal of Obstetrics & Gynecology and Reproductive Biology*.

[18] Levi R., Lundberg U., Hanson U., Frankenhacuser M. (1989). Anxiety during pregnancy after the Chernobyl accident as related to obstetric outcome. *Journal of Psychosomatic Obstetrics and Gynecology*.

[19] Goldenberg R. L., Cliver S. P., Mulvihill F. X. (1996). Medical, psychosocial, and behavioral risk factors do not explain the increased risk for low birth weight among black women. *American Journal of Obstetrics and Gynecology*.

[20] Perkin M. R., Bland J. M., Peacock J. L., Anderson H. R. (1993). The effect of anxiety and depression during pregnancy on obstetric complications. *British Journal of Obstetrics and Gynaecology*.

[21] Rauchfuss M., Maier B. (2011). Biopsychosocial predictors of preterm delivery. *Journal of Perinatal Medicine*.

[22] Martini J., Knappe S., Beesdo-Baum K., Lieb R., Wittchen H.-U. (2010). Anxiety disorders before birth and self-perceived distress during pregnancy: associations with maternal depression and obstetric, neonatal and early childhood outcomes. *Early Human Development*.

[23] Amiri F., Mohamadpour R., Salmalian H., Ahmadi A. (2010). The association between prenatal anxiety and spontaneous preterm birth and low birth weight. *Iranian Red Crescent Medical Journal*.

[24] Andersson L., Sundström-Poromaa I., Wulff M., Åström M., Bixo M. (2004). Neonatal outcome following maternal antenatal depression and anxiety: a population-based study. *American Journal of Epidemiology*.

[25] Berle J. Ø., Mykletun A., Daltveit A. K., Rasmussen S., Holsten F., Dahl A. A. (2005). Neonatal outcomes in offspring of women with anxiety and depression during pregnancy. *Archives of Women's Mental Health*.

[26] Bindt C., Guo N., Te Bonle M. (2013). No association between antenatal common mental disorders in low-obstetric risk women and adverse birth outcomes in their offspring: results from the CDS study in Ghana and Côte D'Ivoire. *PLoS ONE*.

[27] Catov J. M., Abatemarco D. J., Markovic N., Roberts J. M. (2010). Anxiety and optimism associated with gestational age at birth and fetal growth. *Maternal and Child Health Journal*.

[28] Catov J. M., Flint M., Lee M., Roberts J. M., Abatemarco D. J. (2014). The relationship between race, inflammation and psychosocial factors among pregnant women. *Maternal and Child Health Journal*.

[29] Copper R. L., Goldenberg R. L., Das A. (1996). The preterm prediction study: maternal stress is associated with spontaneous preterm birth at less than thirty-five weeks' gestation. *American Journal of Obstetrics and Gynecology*.

[30] Dayan J., Creveuil C., Marks M. N. (2006). Prenatal depression, prenatal anxiety, and spontaneous preterm birth: a prospective cohort study among women with early and regular care. *Psychosomatic Medicine*.

[31] Dole N., Savitz D. A., Siega-Riz A. M., Hertz-Picciotto I., McMahon M. J., Buekens P. (2004). Psychosocial factors and preterm birth among African American and white women in central North Carolina. *American Journal of Public Health*.

[32] Dominguez T. P., Dunkel-Schetter C., Glynn L. M., Hobel C., Sandman C. A. (2008). Racial differences in birth outcomes: the role of general, pregnancy, and racism stress. *Health Psychology*.

[33] Dominguez T. P., Schetter C. D., Mancuso R., Rini C. M., Hobel C. (2005). Stress in African American pregnancies: testing the roles of various stress concepts in prediction of birth outcomes. *Annals of Behavioral Medicine*.

[34] Field T., Diego M., Hernandez-Reif M. (2010). Comorbid depression and anxiety effects on pregnancy and neonatal outcome. *Infant Behavior and Development*.

[35] Glynn L. M., Dunkel-Schetter C., Hobel C. J., Sandman C. A. (2008). Pattern of perceived stress and anxiety in pregnancy predicts preterm birth. *Health Psychology*.

[36] Hosseini S. M., Biglan M. W., Larkby C., Brooks M. M., Gorin M. B., Day N. L. (2009). Trait anxiety in pregnant women predicts offspring birth outcomes. *Paediatric and Perinatal Epidemiology*.

[37] Ibanez G., Charles M.-A., Forhan A. (2012). Depression and anxiety in women during pregnancy and neonatal outcome: data from the EDEN mother-child cohort. *Early Human Development*.

[38] Kramer M. S., Lydon J., Séguin L. (2009). Stress pathways to spontaneous preterm birth: the role of stressors, psychological distress, and stress hormones. *American Journal of Epidemiology*.

[39] Latendresse G., Ruiz R. J. (2011). Maternal corticotropin-releasing hormone and the use of selective serotonin reuptake inhibitors independently predict the occurrence of preterm birth. *Journal of Midwifery and Women's Health*.

[40] Lobel M., Cannella D. L., Graham J. E., DeVincent C., Schneider J., Meyer B. A. (2008). Pregnancy-specific stress, prenatal health behaviors, and birth outcomes. *Health Psychology*.

[41] Lobel M., DeVincent C. J., Kaminer A., Meyer B. A. (2000). The impact of prenatal maternal stress and optimistic disposition on birth outcomes in medically high-risk women. *Health Psychology*.

[42] Mancuso R. A., Schetter C. D., Rini C. M., Roesch S. C., Hobel C. J. (2004). Maternal prenatal anxiety and corticotropin-releasing hormone associated with timing of delivery. *Psychosomatic Medicine*.

[43] McDonald S. W., Kingston D., Bayrampour H., Dolan S. M., Tough S. C. (2014). Cumulative psychosocial stress, coping resources, and preterm birth. *Archives of Women's Mental Health*.

[44] Orr S. T., Reiter J. P., Blazer D. G., James S. A. (2007). Maternal prenatal pregnancy-related anxiety and spontaneous preterm birth in Baltimore, Maryland. *Psychosomatic Medicine*.

[45] Pagel M. D., Smilkstein G., Regen H., Montano D. (1990). Psychosocial influences on new born outcomes: a controlled prospective study. *Social Science and Medicine*.

[46] Peacock J. L., Bland J. M., Anderson H. R. (1995). Preterm delivery: effects of socioeconomic factors, psychological stress, smoking, alcohol, and caffeine. *British Medical Journal*.

[47] Powell H., McCaffery K., Murphy V. E. (2013). Psychosocial variables are related to future exacerbation risk and perinatal outcomes in pregnant women with asthma. *Journal of Asthma*.

[48] Rini C. K., Dunkel-Schetter C., Wadhwa P. D., Sandman C. A. (1999). Psychological adaptation and birth outcomes: the role of personal resources, stress, and sociocultural context in pregnancy. *Health Psychology*.

[49] Sanchez S. E., Puente G. C., Atencio G. (2013). Risk of spontaneous preterm birth in relation to maternal depressive, anxiety, and stress symptoms. *Journal of Reproductive Medicine*.

[50] Wadhwa P. D., Sandman C. A., Porto M., Dunkel-Schetter C., Garite T. J. (1993). The association between prenatal stress and infant birth weight and gestational age at birth: a prospective investigation. *American Journal of Obstetrics and Gynecology*.

[51] Dayan J., Creveuil C., Herlicoviez M. (2002). Role of anxiety and depression in the onset of spontaneous preterm labor. *American Journal of Epidemiology*.

[52] McDowell I. (2006). *Measuring Health: A Guide to Rating Scales and Questionnaires*.

[53] Alipour Z., Lamyian M., Hajizadeh E. (2012). Anxiety and fear of childbirth as predictors of postnatal depression in nulliparous women. *Women and Birth*.

[54] Arch J. J. (2013). Pregnancy-specific anxiety: which women are highest and what are the alcohol-related risks?. *Comprehensive Psychiatry*.

[55] Hall W. A., Stoll K., Hutton E. K., Brown H. (2012). A prospective study of effects of psychological factors and sleep on obstetric interventions, mode of birth, and neonatal outcomes among low-risk British Columbian women. *BMC Pregnancy and Childbirth*.

[56] Spielberger C. D., Gorsuch R. L., Lushene R. E. (1970). *Manual for the State-Trait Anxiety Inventory*.

[57] Spielberger C. D., Vagg P. R. (1984). Psychometric properties of the STAI: a reply to Ramanaiah, Franzen, and Schill. *Journal of Personality Assessment*.

[58] Alder J., Fink N., Bitzer J., Hösli I., Holzgreve W. (2007). Depression and anxiety during pregnancy: a risk factor for obstetric, fetal and neonatal outcome? A critical review of the literature. *Journal of Maternal-Fetal and Neonatal Medicine*.

[59] Borenstein M., Hedges L. V., Higgins J. P. T., Rothstein H. R. (2009). *Introduction to Meta-Analysis*.

[60] Thompson S. G. (1994). Why sources of heterogeneity in meta-analysis should be investigated. *British Medical Journal*.

[61] Lau J., Ioannidis J. P. A., Terrin N., Schmid C. H., Olkin I. (2006). The case of the misleading funnel plot. *British Medical Journal*.

[62] Tang J.-L., Liu J. L. (2000). Misleading funnel plot for detection of bias in meta-analysis. *Journal of Clinical Epidemiology*.

[63] Hill A. B. (1965). The environment and disease: association or causation?. *Proceedings of the Royal Society of Medicine*.

[64] Blencowe H., Cousens S., Chou D. (2013). Born Too Soon: the global epidemiology of 15 million preterm births. *Reproductive Health*.

