# Supplementary material for: Prenatal Maternal Anxiety as a Risk Factor for Preterm Birth and the Effects of Heterogeneity on This Relationship: A Systematic Review and Meta-Analysis
Source: Biomed Res Int. 2016 May 19;2016:8312158. doi: 10.1155/2016/8312158 (PMC4889802; doi:10.1155/2016/8312158)
Supplement: Supplementary file 1 — The Table in the supplementary material presents the details of the critical appraisal according to the Critical Appraisal Skills Program (CASP) tool in which the quality and validity of each study is assessed. The nine CASP questions addressing the potential for bias are provided in Table 1 of the manuscript and each is answered with a Yes, Can't tell or No. [file 8312158.f1.pdf]

Supplement 1 Association between maternal vitamin D status and maternal toll-like receptor triggered cytokine response

	Univariate analysis		Multiviariate analysis	
	β (95% CI)	p	β (95% CI)	p
TLR1-2				
TNF-α	0.06 (-0.003, 0.12)	.06	0.05 (−0.02, 0.12)	.14
IL-6	−0.07 (−0.16, 0.03)	.15	−0.08 (−0.18, 0.03)	.16
IL-10	−0.01 (−0.19, 0.18)	.95	0.12 (−0.19, 0.43)	.39
TLR3				
TNF-α	0.03 (−0.02, 0.07)	.24	0.23 (−0.02, 0.07)	.32
IL-6	0.01 (−0.05, 0.06)	.85	0.01 (−0.05, 0.07)	.72
IL-10	−0.15 (−0.35, 0.06)	.14	−0.04 (−0.45, 0.37)	.83
TLR4				
TNF-α	−0.004 (−0.09, 0.08)	.92	−0.08 (−0.10, 0.08)	.85
IL-6	−0.009 (−0.13, 0.11)	.88	0.003 (−0.12, 0.13)	.96
IL-10	0.63 (0.12, 1.13)	.02	0.66 (−0.11, 1.43)	.08
TLR7-8				
TNF-α	0.02 (−0.05, 0.08)	.64	0.03 (−0.04, 0.10)	.43
IL-6	0.01 (−0.07, 0.08)	.99	−0.03 (−0.12, 0.06)	.51
IL-10	−0.04 (−0.32, 0.25)	.79	−0.30 (−0.93, 1.60)	.57
PHA				
TNF-α	0.03 (−0.06, 0.11)	.53	0.004 (−0.09, 0.10)	.94
IL-6	−0.03 (−0.10, 0.04)	.46	−0.03 (−0.11, 0.05)	.41
IL-10	0.02 (−0.15, 0.19)	.81	−0.07 (−0.32, 0.19)	.58

Number of samples: (TNF: 47; Il-6: 41; IL-10: 28)
Adjusted for gestational age, gender, birth body weight, mode of delivery, maternal allergy, and season of birth

Supplement 2 Relation of maternal and cord blood 25(OH)D to TLR9-triggered cytokine response

	Univariate analysis β (95% CI)	p	Multivariate analysis β (95% CI)	p
Maternal 25(OH)D				
TNF-α	0.11 (-0.07, 0.30)	.18	0.29 (-0.48 - 1.06)	.13
IL-6	0.00 (-0.07, 0.67)	.98	0.03 (-0.14 – 0.19)	.65
IL-10	-0.08 (-0.23, 0.08)	.28	-0.24 (-0.79 – 0.30)	.25
Cord 25(OH)D				
TNF-α	-0.10 (-0.24, 0.04)	.14	-0.12 (-0.38, 0.14)	.23
IL-6	-0.05 (-0.12, 0.02)	.12	-0.06 (-0.15, 0.02)	.12
IL-10	-0.16 (-0.31, -0.01)	.04	-0.20 (-0.11, 0.07)	.41

Number of samples: (TNF: 18; Il-6: 20; IL-10: 17)
Adjusted for gestational age, parity, gender, birth body weight, mode of delivery, and maternal allergy
Both 25(OH)D and cytokine level were logarithmically transformed for analysis as continuous variables
